# Metabolome analysis revealed that soybean–*Aspergillus oryzae* interaction induced dynamic metabolic and daidzein prenylation changes

**DOI:** 10.1371/journal.pone.0254190

**Published:** 2021-07-02

**Authors:** Haruya Takahashi, Koji Ochiai, Kuni Sasaki, Atsushi Izumi, Yu Shinyama, Shinsuke Mohri, Wataru Nomura, Huei-Fen Jheng, Teruo Kawada, Kazuo Inoue, Tsuyoshi Goto

**Affiliations:** 1 Laboratory of Molecular Function of Food, Division of Food Science and Biotechnology, Graduate School of Agriculture, Kyoto University, Kyoto, Japan; 2 DAIZ Inc., Kumamoto, Japan; 3 Research Unit for Physiological Chemistry, Kyoto University, Kyoto, Japan; 4 National Applied Research Laboratories, National Laboratory Animal Center, Taipei, Taiwan; Institute for Biological Research, SERBIA

## Abstract

Several isoflavonoids are well known for their ability to act as soybean phytoalexins. However, the overall effects of the soybean–*Aspergillus oryzae* interaction on metabolism remain largely unknown. The aim of this study is to reveal an overview of nutritive and metabolic changes in germinated and *A*. *oryzae*-elicited soybeans. The levels of individual nutrients were measured using the ustulation, ashing, Kjeldahl, and Folch methods. The levels of individual amino acids were measured using high-performance liquid chromatography. Low-molecular-weight compounds were measured through metabolome analysis using liquid chromatography-mass spectrometry. Although the levels of individual nutrients and amino acids were strongly influenced by the germination process, the elicitation process had little effect on the change in the contents of individual nutrients and amino acids. However, after analyzing approximately 700 metabolites using metabolome analysis, we found that the levels of many of the metabolites were strongly influenced by soybean–*A*. *oryzae* interactions. In particular, the data indicate that steroid, terpenoid, phenylpropanoid, flavonoid, and fatty acid metabolism were influenced by the elicitation process. Furthermore, we demonstrated that not the germination process but the elicitation process induced daidzein prenylation, suggesting that the soybean–*A*. *oryzae* interactions produce various phytoalexins that are valuable for health promotion and/or disease prevention.

## Introduction

Soybean (*Glycine max*) is an essential food, forage, and bioenergy crop. In Japan, soybean is commonly used as a source of food, forage, and energy. Soybeans contain abundant energy sources, including proteins and lipids. Therefore, they are a source of plant-derived protein and edible oils in both human diets and livestock forage [1]. In addition to caloric content (energy) soybeans also contain food additives and bioactive substances. Soy isoflavones act as phytoestrogens [2]. Recent study using gas chromatography–mass spectrometry (GC–MS) and liquid chromatography–MS (LC–MS) has reported that germination process in soybean induces changes in amino acids and isoflavones associated with health benefits and/or taste quality [3].

Plant defense against fungal pathogens induces the generation of various secondary metabolites, including phytoalexins, which are partially beneficial for health. The purpose of the present study was to analyze the interactions between the host plant (in this case, *Glycine max*) and the associated fungus (*Aspergillus oryzae*), in particular the host plant’s defense against disease. Plant diseases induced by fungi are a significant threat to economic and food security. The interaction between fungi and plants induces a wide array of biological processes [4]. Metabolic changes, including the synthesis of phytoalexins and lignin, are involved in plant defense against fungal pathogens [5]. Previous studies have reported that the levels of phenylpropanoids, flavonoids, and fumaric acid in plants were increased following infection by fungal pathogens [6]. Phytoalexin degradation by pathogens is essential for disease development [7]. Some phytoalexins are beneficial for health. Prenylated phenolics from legume seedlings can serve multiple purposes; for instance, as phytoestrogens, prenylated phenolics provide health benefits and serve as natural antimicrobials for food preservation [8]. Furthermore, glyceollins, another type of phytoalexin, show activities that are beneficial to human health, including anticancer effects [9]. Recently, it was reported that the interactions between soybean and *Rhizopus microsporus var*. *oryzae* increases the content and diversity of isoflavonoids, including glyceollins [10]. Therefore, studies of plant-fungal interactions are crucial for preventing plant diseases and producing bioactive substances.

Although metabolomic analysis has been performed to investigate plant–fungal interactions, little is known regarding the effects of *A*. *oryzae* on metabolism in soybean. In metabolomic analysis, ultra-accurate mass spectrometry is used along with a metabolic information database. Metabolomic analyses enable the identification of molecules based on the exact mass data and annotations retrieved from a metabolite database [11–14]. Over the past decade, metabolomic analyses based on liquid chromatography–mass spectrometry (LC-MS), gas chromatography–mass spectrometry (GC-MS), and other analytical techniques have been applied to investigate the interactions between plants and fungi [4]. Although metabolomic analysis has been used to investigate the interactions between soybean and several fungal pathogens [10–19], little is known about the interaction between soybean and *A*. *oryzae*. In a previous study, 12 isoflavones were identified through LC-MS, and their content was reported to change in a time-dependent manner following *A*. *oryzae* stimulation [19]. However, little is known about how the interactions between soybean and *A*. *oryzae* affect the production of metabolites, including phytoalexins.

In the present study, we aimed to provide an overview of nutritive and metabolic change in germinated and *A*. *oryzae*-elicited soybean and detect metabolites influenced by the interactions between soybean and *A*. *oryzae*. The final purpose was to better understand the effect of *A*. *oryzae* stimulation on soybean metabolism.

## Materials and methods

### Materials

Soybean seeds (*Glycine max L*. ‘Yukipirika’; raw seeds, 200 g dry weight) were placed into a container (220 × 158 × 81 mm^3^), which could control the drain rate. The seeds in the container were grown in a germination chamber (SU Technos, Kumamoto, Japan) at 28°C and water at intervals of 30 min. After 72 h, the germinated seeds were inoculated with 10 g of *A*. *oryzae* AOK139 strain (Akita Konno Co., Ltd.). The control germinated seeds (“germination” group) or elicited germination seeds (“elicitation” group) were incubated under high humidity without watering. After 96 h of elicitation, samples were randomly collected from intact seeds that expressed hypersensitive cell death; the samples were vacuum-packed and stored at -80°C.

All chemicals were obtained from Wako (Osaka, Japan), Nacalai Tesque (Kyoto, Japan), and Sigma-Aldrich (St. Louis, MO, USA). The buffers used were of high-performance liquid chromatography (HPLC) or LC-MS grade.

### Quantification of water and nutrient (carbohydrates, lipids, minerals, and proteins) content

Water, mineral, protein, and lipid content of the raw seed, germination, and elicitation samples were measured by the ustulation, ashing, Kjeldahl, and Folch methods, respectively. In the ustulation method, the water content was calculated as the difference in weight before and after heating using a dryer. In the ashing method, the mineral content was calculated as the difference in weight between before and after heating using an electric muffle furnace (550°C). In the Kjeldahl method, the sample was heated with concentrated sulfuric acid. The amount of nitrogen in the sample is reflected in the ammonium ion concentration in the acid solution and this concentration is measured via titration. The protein amount was obtained by multiplying the amount of nitrogen with 6.25 (conversion factor of nitrogen-protein). Lipids were extracted using a chloroform/methanol solution (2:1, v/v). The extract was evaporated under vacuum using a rotary evaporator. The residue was lysed using petroleum ether and anhydrous sodium sulfate. After eliminating the solvent was with a dryer, the residue was collected as a lipid. Carbohydrate content was calculated using the following equation:

C.C.=100–W.C.–L.C.–M.C.–P.C.

where C.C is the carbohydrate content (g·100 g^-1^), W.C. is the water content (g·100 g^-1^), L.C. is the lipid content (g·100 g^-1^), M.C. is the mineral content (g·100 g^-1^), and P.C. is the protein content (g·100 g^-1^).

### Quantification of amino acids

HPLC-based amino acid analysis and sample extraction were performed as described previously [20] with some modifications. Amino acids in raw seeds, germination, and elicitation samples were extracted using 70% ethanol. HPLC was performed using a Nexera X2 HPLC system (Shimadzu, Kyoto, Japan) coupled with an automated pre-column derivatization functionality and a fluorescence detector (excitation wavelength, 266 nm; emission wavelength, 305 nm). The standard amino acids (type H) and GABA were diluted 5% HCl.

In the derivatization, boric acid solution was prepared by dissolving boric acid (0.31 g) and NaOH (0.1 g) in diluted water (50 mL). 3-Mercaptopropionic Acid (MPA) solution were prepared by dissolving 10 μL of MPA in boric acid solution (10 mL). *O*-phthalaldehyde (OPA) solution was prepared by dissolving OPA (10 mg) in ethanol (0.3 mL), boric acid solution (0.7 mL) and diluted water (4 mL). 9-fluorenylmethyl chloroformate (FMOC) solution was prepared by dissolving FMOC (10 mg) in acetonitrile (25 mL). At first, the extracted solution containing amino acids or the standard amino acid solutions was mixed with OPA and MPA solutions (1:3:6, v/v/v) and standing (1min). Next, the solution was mixed with FMOC solution (10:1, v/v) and standing (2 min).

An aliquot of the sample was injected into an Inertsil ODS-4HP reversed-phase column (column size, 3.0 × 100 mm; particle size, 3.0 μm; GL Sciences Inc., Tokyo, Japan). Mobile phases A (15 mM potassium dihydrogen phosphate/ 5 mM potassium hydrogen phosphate buffer) and B (water: acetonitrile: methanol = 15:45:40, v/v/v) were used. The buffer gradient was as follows: 9.5% B for 0.0 to 1.5 min, 9.5 to 30.0% B for 1.5 to 6.0 min, 30.0 to 40.0% B for 6.0 to 11.0 min, 40.0 to 100.0% B for 11.0 to 16.07 min, 100.0% B for 16.07 to 36.0 min, and 100.0 to 9.5% B for 36.0 to 36.5 min; the flow rate was 800 μL·min^-1^. Data including peak detection were acquired using LabSolutions software (Shimadzu, Kyoto, Japan).

### Metabolomic analysis using LC-MS

LC-MS-based metabolomic analysis and sample extraction were performed as described previously [21, 22] with some modifications. Low-molecular weight compounds in raw seeds, germination, and elicitation samples were extracted using 80% methanol. LC-MS was performed using an HPLC system (Ultimate 3000 RSLC) coupled to a Q Exactive Orbitrap-MS system (Thermo Fisher Scientific, CA, USA) equipped with an electrospray source operating in the positive and negative ion modes (m/z range: 80–1,200). The capillary and gas heater temperature were set at 300°C and 400°C, respectively. The spray voltage was set at 3.2 kV (positive ion mode) or 2.5 kV (negative ion mode). The precursor ions selected by data dependent scan (top 10 ions with most intensity/ scan) were measured with the use of MS^2^ scan mode. An aliquot of the extracted sample was injected into the InertSustain AQ-C18 reversed-phase column (column size, 2.1 × 150 mm; particle size, 3.0 μm). Mobile phases A (0.1% formic acid) and B (acetonitrile) were used. The buffer gradient was as follows: 2.0% B for 0.0 to 3.0 min, 2.0 to 98.0% B for 3.0 to 30.0 min, 98.0% B for 30.0 to 35.0 min, 98.0 to 2.0% B for 35.0 to 35.1 min, and 2.0% B for 4.9 min before the next injection. The flow rate was 200 μL·min^-1^. Data were acquired using Compound Discoverer 2.1 (Thermo Fisher Scientific) linked to the Kyoto Encyclopedia of Genes and Genomes (KEGG) and mzCloud^TM^ databases. Compound Discoverer 2.1 is a software package for detection and annotation of peaks obtained using LC-MS. The minimum peak intensity was set at 10,000. The value of peak area was used to calculate the rate of change between different groups. Differences between groups were compared with Student’s *t*-test. In the processing of predictive composition and KEGG search, the mass tolerance was set at 5 ppm. In the processing of mzCloud search, the precursor mass tolerance and the fragment mass tolerance were set at 10 ppm and 0.4 Da, respectively. The above-mentioned method was used to identify neobavaisoflavone in the elicitation sample.

### Statistical analysis

Data are presented as mean ± SEM. Data were analyzed using Student’s *t*-test (for two groups) or multiple comparison tests (Tukey test). Differences were considered significant at *p* < 0.05.

## Results

### Effect of germination and elicitation on the water and nutrient content of soybean

**Fig 1:**
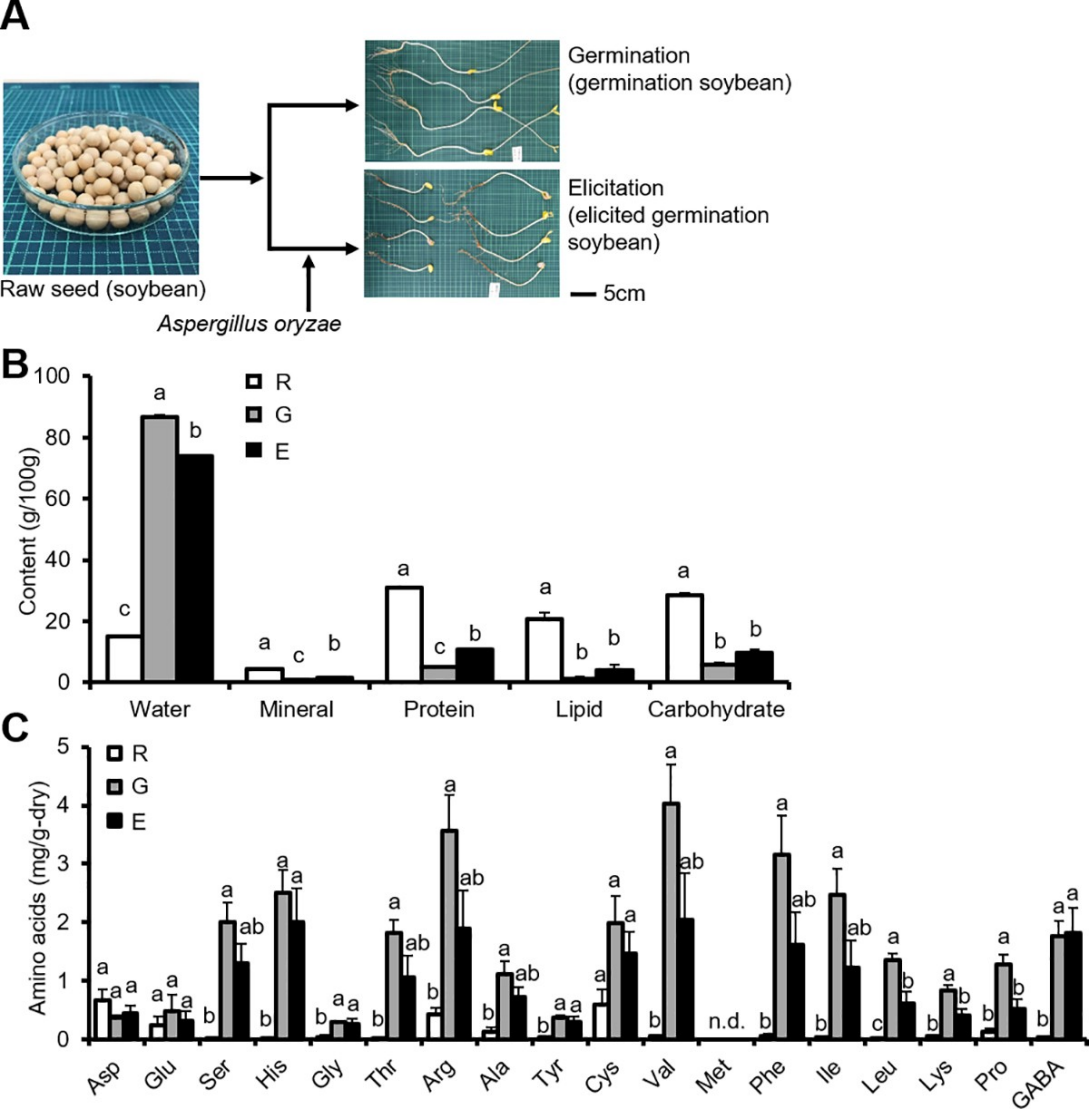
Effects of germination and elicitation on water and nutrient content of soybean. **(A)** Processes of germination and elicitation in soybean. **(B)** Water, protein, lipid, mineral, and carbohydrate content of raw seed, germination, and elicitation samples. **(C)** Amino acid content of raw seed, germination, and elicitation samples. Data are expressed as means ± SEM (B: n = 5 /group; C: n = 3 /group). Different letters indicate significant differences (*p* < 0.05). R: raw seed (soybean); G: germination (germinated soybean); E: elicited germinated soybean. n.d.: not detected.

First, raw seed (soybean), germination (germinated soybean), and elicitation (elicited germinated soybean) samples were prepared using the above-mentioned methods (Fig 1A). During germination, protein, lipid, mineral, and carbohydrate content decreased (Fig 1B) whereas the water content increased (Fig 1B). Meanwhile, during elicitation, protein and mineral content increased (Fig 1B), whereas water content decreased (Fig 1B). Elicitation did not affect lipid and carbohydrate content (Fig 1B). There were significant differences in the contents of most detected amino acids, except Asp, Glu, and Cys, between the raw seed and germination samples (dry) (Fig 1C). There were no differences in the contents of most detected amino acids, except Leu, Lys, and Pro, between the germination and elicitation samples (dry) (Fig 1C).

### Comparative metabolomic analysis of the raw seed, germinated, and elicited germinated samples

**Fig 2:**
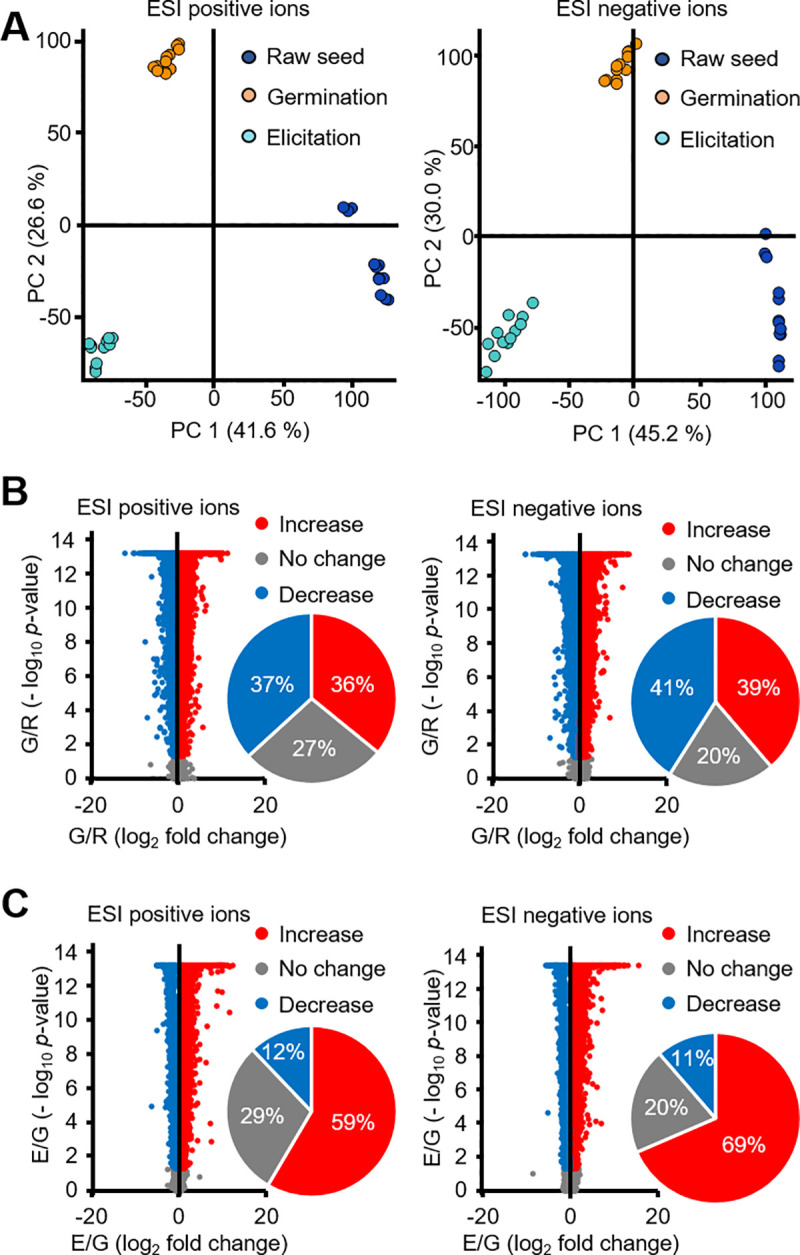
PCA and volcano plot analysis of the detected MS peaks in raw seed, germination, and elicitation samples. **(A)** PCA of raw seed, germination, and elicitation samples. Volcano plots and comparison of change ratios between **(B)** raw seed and germination samples and **(C)** germination and elicitation samples.

In LC-MS-based metabolomic analysis, PCA revealed that the detected peaks were markedly different among the raw seed, germination, and elicitation samples (Fig 2A). Comparison of the raw seed and germination samples showed that 36% of the detected electrospray ionization (ESI)-positive and 39% of the ESI-negative ion peaks were significantly increased in raw seed samples, whereas 37% of the detected ESI-positive and 41% of the ESI-negative ion peaks were significantly decreased in the germinated samples (Fig 2B). Comparison of the germination and elicited germinated samples showed that 59% and 69% of the detected ESI-positive and negative ion peaks, respectively, were significantly increased in the germinated samples, but 12% of the detected ESI-positive and 11% of the ESI-negative ion peaks were significantly decreased in the germinated samples (Fig 2C).

**Fig 3:**
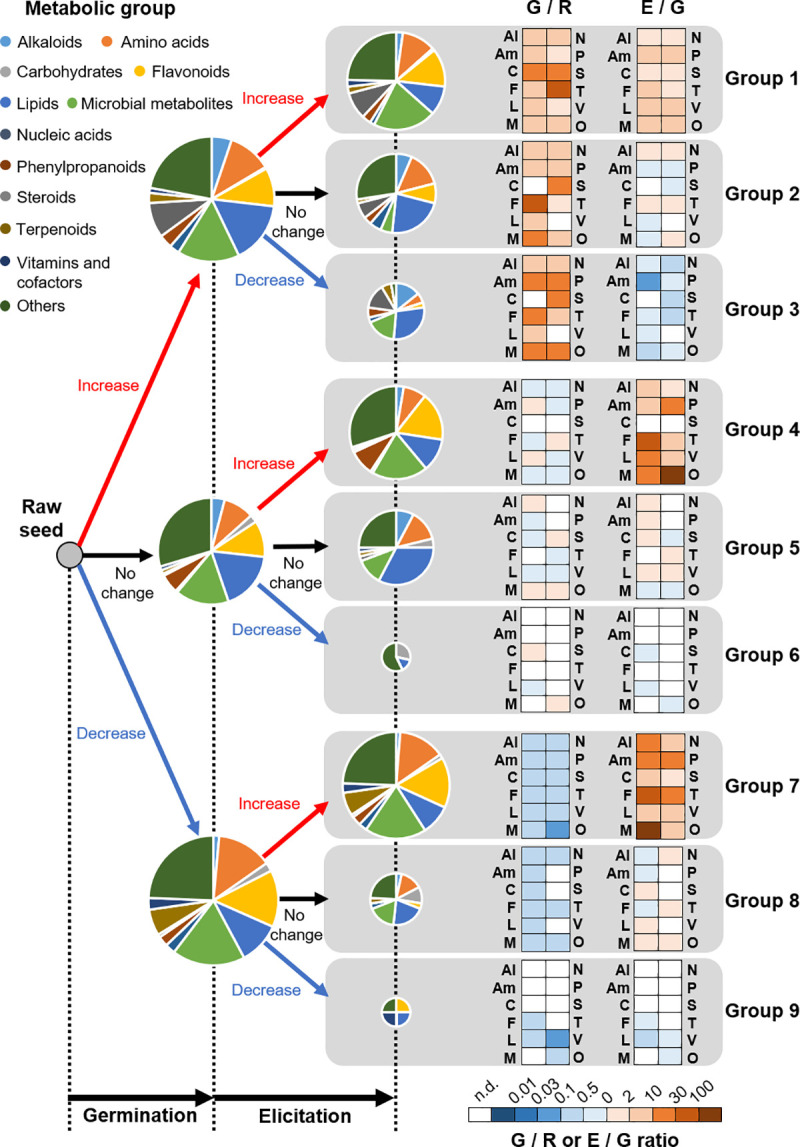
Estimated effects of germination and elicitation on the metabolic profile of raw seed, germination, and elicitation samples. The pie chart and heat map represent the proportion and average change ratios of individual metabolic groups, respectively. The size of the pie chart represents the number of estimated metabolites (appended under arrows). Metabolic groups: Al, alkaloid; Am, amino acid; C, carbohydrate; F, flavonoid; L, lipid; M, microbial metabolite; N, nucleic acid; P, phenylpropanoid; S, steroid group; T, terpenoid group; V, vitamin and cofactor group; O, other. Details of the metabolites are given in S1 Table.

Next, the estimated compound names corresponding to the detected peaks were annotated using the KEGG database based on the exact mass data. The molecular formula was estimated using the detected exact mass. We linked the estimated molecular formula data to metabolic information from the KEGG database. Using KEGG compound and pathway information, the estimated molecular formula data were classified in the following groups: ‘Alkaloids’, ‘Amino acids’, ‘Carbohydrates’, ‘Flavonoids’, ‘Lipids’, ‘Microbial metabolites’, ‘Nucleic acids’, ‘Phenylpropanoids’, ‘Steroids’, ‘Terpenoids’, ‘Vitamins and cofactors’, and ‘Others’ group. A total of 678 compounds were classified into 12 metabolic groups (Al, Am, C, F, L, M, N, P, S, T, V, and O groups; Fig 3 and S1 Table) and nine patterns of change between raw and germinated soybean as well as between germinated and elicited germinated soybean (groups 1–9; Fig 3 and S1 Table). With the exception microbial and other metabolic groups, the content and number of flavonoid group metabolites were the highest in group 1 (12.7%, 17 metabolites), 4 (16.8%, 19 metabolites), and 7 (15.3%, 37 metabolites), whose levels were increased by elicitation (Fig 3, pie chart). The average change ratios of these flavonoid group metabolites due to germination and elicitation were 3.1- and 9.1-fold in group 1, 0.9- and 94.4-fold in group 4, and 0.3- and 35.0-fold in group 7, respectively (Fig 3, heat map).

### Metabolites induced by *A*. *oryzae*-elicited germination

**Fig 4:**
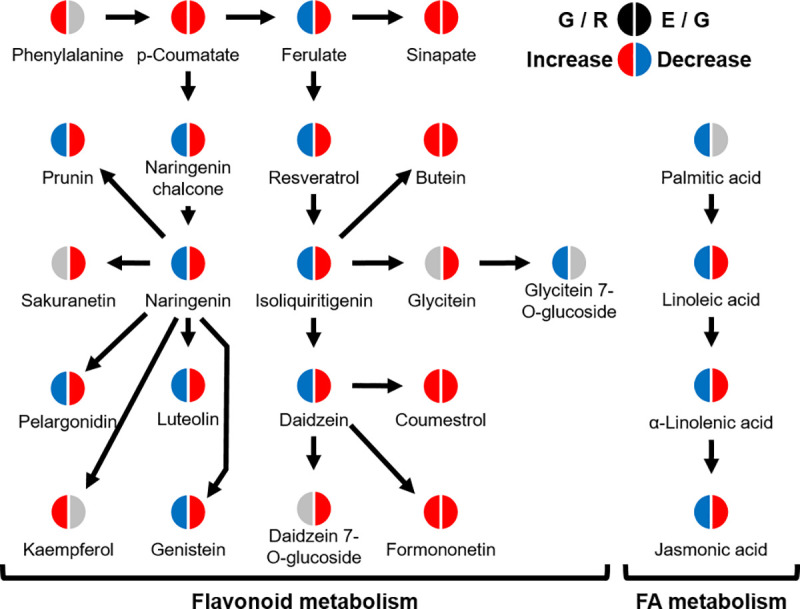
Comparative analysis of metabolic pathways among raw seed, germination, and elicitation samples. The estimated effect of germination and elicitation on flavonoid and fatty acid metabolic pathways. Details of these metabolites are given in S2 Table and S1 Fig.

For precision analysis, MS^2^ spectra of the detected metabolites in soybean were collated with the mzCloud compounds database. The detected MS^2^ spectra data were linked to the MS^2^ spectra data of authentic compounds stored in the mzCloud library database. The compound name was estimated using both the molecular formula data and MS^2^ spectra data (S2 Table and S1 Fig). Metabolic pathway analysis using the MS^2^ spectrum database suggested that parts of flavonoid and fatty acid metabolism were inhibited by germination but promoted by elicitation (Fig 4).

**Fig 5:**
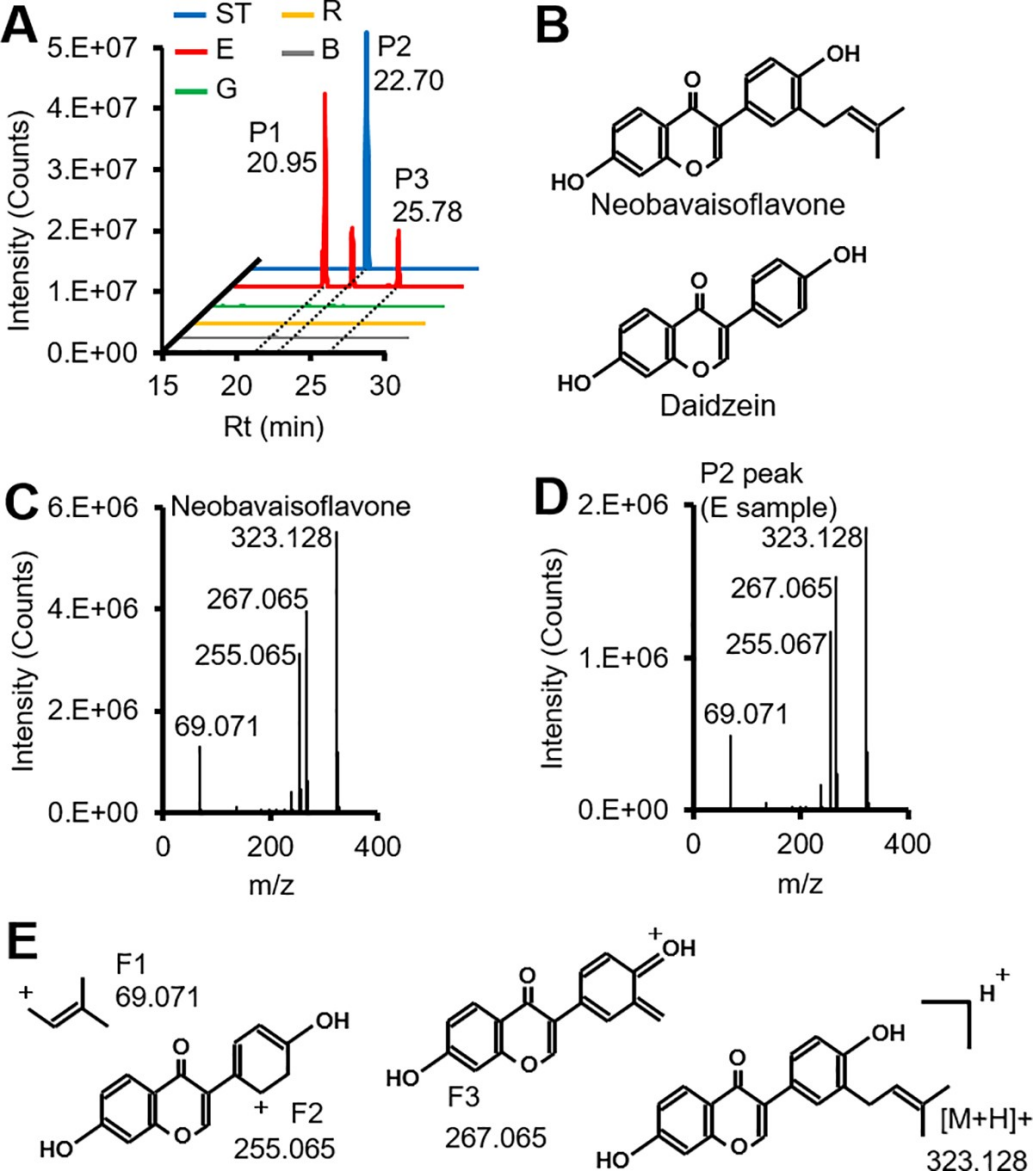
Identification of neobavaisoflavone in *A*. *oryzae*-elicited germinated soybean. **(A)** Extracted ion chromatogram of m/z = 323.1278. ST: standard (neobavaisoflavone, 100 pg); R: raw seed (soybean); G: germination (germinated soybean); E: elicited germinated soybean; B: blank. **(B)** Structural formulae of neobavaisoflavone and daidzein. MS^2^ spectra of **(C)** neobavaisoflavone and **(D)** P2 peak (E sample). **(E)** Structural formulas of neobavaisoflavone fragments (F1-3) and H^+^ adduct ion ([M+H]^+^).

In addition, we found that three peaks of m/z = 323.1278 (P1-3) were specific to germinated soybean (Fig 5A), and these exact masses corresponded to neobavaisoflavone (prenylated daidzein, Fig 5B). In the extracted ion chromatogram, the unique peak of neobavaisoflavone corresponded to the P2 peak (Fig 5A). The MS^2^ spectrum of the P2 peak was in acceptable agreement with that of neobavaisoflavone (Fig 5C–5E).

## Discussion

Although the nutrient content of soybean was markedly altered by germination, the effects of elicitation on these changes were definite. Soybean germination increased water content but decreased protein, lipid, mineral, and carbohydrate contents (Fig 1), promoting catabolism. Elicitation increased protein content but decreased Leu, Lys, and Pro content (Fig 1), partially promoting anabolism. However, the increased protein content likely reflects an increase in *A*. *oryzae* proteins. Moreover, we demonstrated that the effects of elicitation on amino acid metabolism were definite. However, it has been previously reported that the content of most α-amino acids in soybean was altered at 24 or 48 h following *Rhizoctonia solani* infection [16]. Further studies are warranted to elucidate the effects of elicitation on amino acid metabolism.

Although the effects of elicitation on changes in the nutrient content of germinated soybean were definite, metabolomic analysis revealed that many metabolites were strongly affected by both germination and elicitation. A large proportion of the detected peaks were altered by germination (ESI-positive ions: 73%, ESI-negative ions: 80%; Fig 2) or elicitation (ESI-positive ions: 71%, ESI-negative ions: 80%; Fig 2). Although previous studies have revealed the effects of elicitation on part of secondary metabolism [8, 16, 19, 23], whether elicitation produces a wide effect on metabolism remains unclear. In the profile data of approximately 700 metabolites predicted by the KEGG database (Fig 3), there were three typical change patterns. Specifically, the levels of metabolites in group 1were increased by both germination and elicitation, those of metabolites in group 4 were increased by elicitation alone, and those of metabolites in group 7 were decreased by germination but increased by elicitation. Groups 1, 4, and 7 contained specific steroids, phenylpropanoids, and terpenoids, respectively (Fig 3). These findings suggest that steroid and terpenoid metabolism is influenced by both germination and elicitation, but phenylpropanoid metabolism is largely influenced by elicitation. A previous study reported that *Rhizoctonia solani* infection induced certain phenylpropanoids in soybean [16], which supports our findings. However, specific metabolites corresponding to these profiles were not identified and instead predicted based on exact mass data using the KEGG database. Further investigations are warranted to explore the effects of germination and elicitation on these secondary metabolic pathways.

Furthermore, our data suggested that the effects of germination on flavonoid and fatty acid metabolism differed from those of elicitation. The MS^2^ data showed that the levels of approximately half of the metabolites that were predicted to be flavonoids were decreased by germination but increased by elicitation (Fig 4). This finding indicates that the effects of germination on flavonoid metabolism differ from those of elicitation. Germination in light tended to increase coumestrol but decrease daidzein and genistein, whereas *Rhizopus oryzae* elicitation tended to increase these three isoflavonoids [23], which is consistent with our findings. Jasmonic acid (JA) is an essential plant hormone in plant–pathogen defense signaling [24], and *Rhizoctonia solani* infection has been reported to upregulate α-linolenic acid and JA expression [16], which is consistent with our findings. It is likely that the different phytoalexin types elicited by different elicitors might be resulted from the phytoalexin detoxification by fungi. In our study, living soybean was used for elicitation and capable of the plant defense reactions. Therefore, comparison of the speed of the phytoalexin detoxification by fungi with that of phytoalexin production by living soybean is difficult. Although it has been suggested that the interaction between living soybean and *A*. *oryzae* induces the dynamic metabolic changes, including phytoalexins, further investigations are needed in order to reveal the mechanism of phytoalexin production and degradation.

We detected neobavaisoflavone in *A*. *oryzae*-elicited germinated soybeans (Fig 5). Neobavaisoflavone is a prenylated daidzein, which was first isolated from *Psoralea corylifolia* [25]. *Psoralea corylifolia* has traditionally been used for the treatment of leucoderma and other skin diseases, pollakiuria, nephritis, asthma, osteoporosis, hypertension, and cardiovascular diseases [26–28]. The mature or dry fruits of this plant are used as health supplements [29]. Neobavaisoflavone exhibits anti-inflammatory, anti-oxidative, and anti-cancer effects [28, 30, 31]. Neobavaisoflavone inhibited melanogenesis through regulating the Akt/GSK-3beta and MEK/ERK pathways in B16F10 cells [32] and inflammatory mediators in activated RAW264.7 macrophages [33]. These findings suggest that neobavaisoflavone may serve as a potent metabolite to promote health and prevent diseases. Our findings pave the way for the use of soybean for health benefits.

The metabolites detected in the present study, except neobavaisoflavone, were not identified but rather predicted based on the KEGG and mzCloud^TM^ databases. Our data suggest the presence of unknown metabolites that influence elicitation. Further investigation is warranted to understand the effects of elicitation on plant metabolism.

In the present study, approximately 700 metabolites in soybean were predicted by metabolomic analysis, and the majority of these metabolites were influenced by *A*. *oryzae* elicitation; however, the effects of elicitation on nutrient content were definite. Our results indicate that the interaction between germinated soybean and *A*. *oryzae* may produce various bioactive substances, which are beneficial for promoting health and preventing diseases.

## Supporting information

S1 Fig MS^2^ spectra of metabolite ‘C01-C25’ are represented by (A)–(Y) data. MS^2^ spectra of the raw file data (C01-C25) and reference data (R01-R25) are presented in the top and bottom panels, respectively. All reference data were obtained from the mzCloud library database. Each annotated metabolic information (C01-C25 and R01-R25) is described in S2 Table. The horizontal axis represents the *m*/*z*.
(PDF)

S1 Table Details of metabolites shown in Fig 3. Annotation was performed using KEGG database. RT: Retention time; R: Raw seed; G: Germination soybean; E: Elicited germination soybean. ACN, acetonitrile; MeOH, methanol; HAc, acetic acid; DMSO, dimethyl sulfoxide; FA, formic acid
(XLSX)

S2 Table Details of metabolites shown in Fig 4. Annotation was performed using the mzCloud library database. RT: Retention time; R: Raw seed; G: Germination soybean; E: Elicited germination. *cf. S1 Fig.
(XLSX)
