# Supplementary figures and images for: Metabolome analysis revealed that soybean–Aspergillus oryzae interaction induced dynamic metabolic and daidzein prenylation changes
Source: PLoS One. 2021 Jul 2;16(7):e0254190. doi: 10.1371/journal.pone.0254190 (PMC8253397; doi:10.1371/journal.pone.0254190)

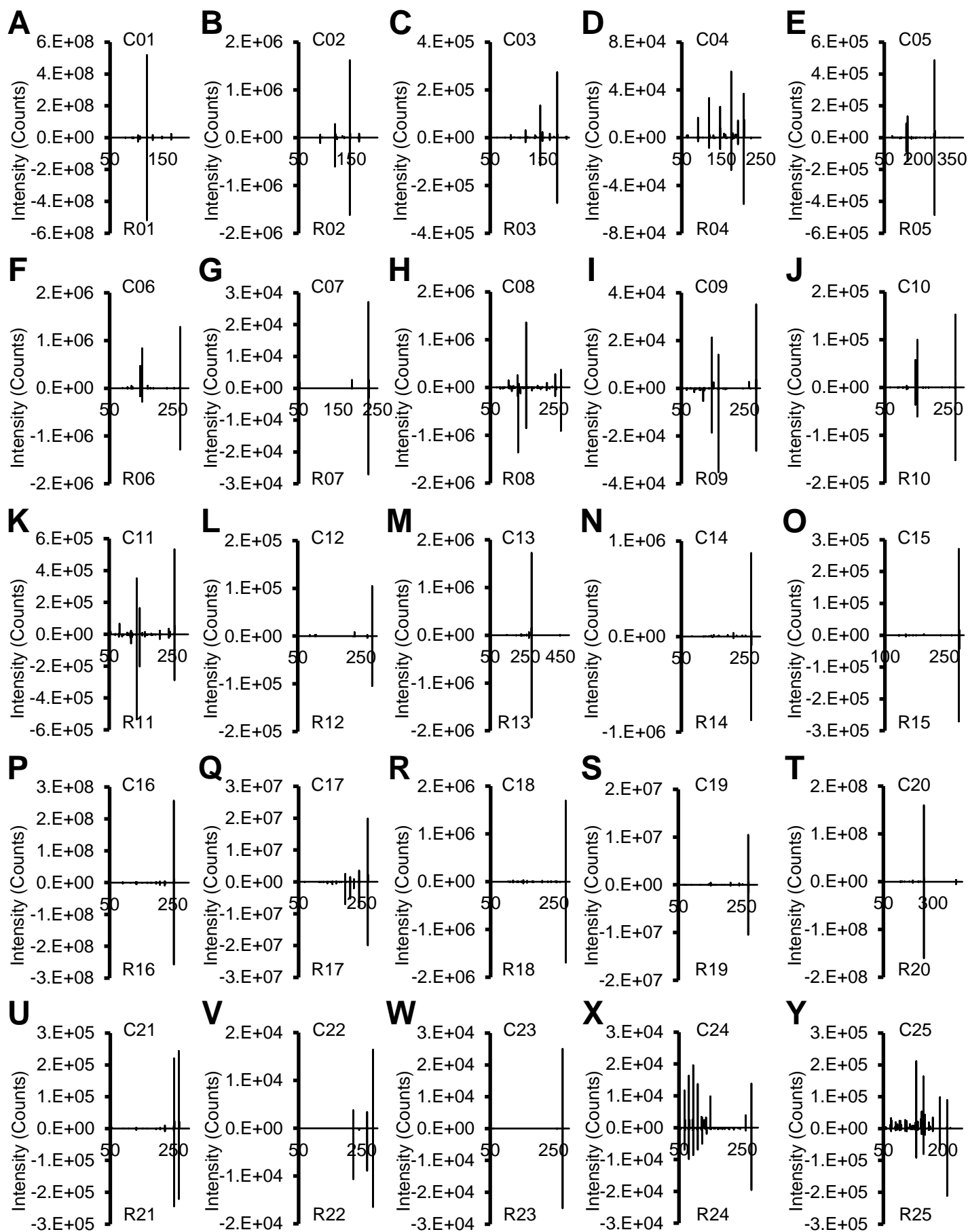

Supplement: S1 Fig — MS2 spectra of the raw file data (C01-C25) and reference data (R01-R25) are presented in the top and bottom panels, respectively. All reference data were obtained from the mzCloud library database. Each annotated metabolic information (C01-C25 and R01-R25) is described in S2 Table. The horizontal axis represents the m/z. (PDF) [file pone.0254190.s001.pdf]
